# Quantitative spectral quality assessment technique validated using intraoperative *in vivo* Raman spectroscopy measurements

**DOI:** 10.1117/1.JBO.25.4.040501

**Published:** 2020-04-21

**Authors:** Frédérick Dallaire, Fabien Picot, Jean-Philippe Tremblay, Guillaume Sheehy, Émile Lemoine, Rajeev Agarwal, Samuel Kadoury, Dominique Trudel, Frédéric Lesage, Kevin Petrecca, Frédéric Leblond

**Affiliations:** a Polytechnique Montréal, Department of Computer Engineering and Software Engineering, Montréal, Québec, Canada; b Centre de Recherche du Centre Hospitalier de l’Université de Montréal, Montréal, Québec, Canada; c Polytechnique Montréal, Department of Engineering Physics, Montréal, Québec, Canada; d Polytechnique Montréal, Department of Electrical Engineering Montréal, Québec, Canada; e ODS Medical Inc., Montréal, Québec, Canada; f Université de Montréal, Department of Pathology and Cellular Biology, Montréal, Québec, Canada; g Centre Hospitalier de l’Université de Montréal, Department of Pathology, Québec, Canada; h Centre de Recherche de l’Institut de Cardiologie de Montréal, Montréal, Québec, Canada; i McGill University, Montreal Neurological Institute and Hospital, Brain Tumour Research Center, Department of Neurology and Neurosurgery, Montréal, Québec, Canada

**Keywords:** Raman spectroscopy, fluorescence, surgery, tissue optics, signal processing, machine learning

## Abstract

**Significance:** Ensuring spectral quality is prerequisite to Raman spectroscopy applied to surgery. This is because the inclusion of poor-quality spectra in the training phase of Raman-based pathology detection models can compromise prediction robustness and generalizability to new data. Currently, there exists no quantitative spectral quality assessment technique that can be used to either reject low-quality data points in existing Raman datasets based on spectral morphology or, perhaps more importantly, to optimize the *in vivo* data acquisition process to ensure minimal spectral quality standards are met.

**Aim:** To develop a quantitative method evaluating Raman signal quality based on the variance associated with stochastic noise in important tissue bands, including C─C stretch, ${\mathrm{CH}}_{2}/{\mathrm{CH}}_{3}$ deformation, and the amide bands.

**Approach:** A single-point hand-held Raman spectroscopy probe system was used to acquire 315 spectra from 44 brain cancer patients. All measurements were classified as either high or low quality based on visual assessment (qualitative) and using a quantitative quality factor (QF) metric. Receiver-operator-characteristic (ROC) analyses were performed to evaluate the performance of the quantitative metric to assess spectral quality and improve cancer detection accuracy.

**Results:** The method can separate high- and low-quality spectra with a sensitivity of 89% and a specificity of 90% which is shown to increase cancer detection sensitivity and specificity by up to 20% and 12%, respectively.

**Conclusions:** The QF threshold is effective in stratifying spectra in terms of spectral quality and the observed false negatives and false positives can be linked to limitations of qualitative spectral quality assessment.

There is growing interest in medicine for systems and methods integrating Raman spectroscopy into clinical workflows to enhance the molecular informational content provided to clinicians.^1^ In particular, over the last decade, there has been significant efforts developing Raman microspectroscopy to complement standard histopathology analyses to improve diagnostic accuracy (e.g., reduce interpathologists variance) and create new avenues to improve disease stratification by providing patient-specific therapeutic options.^2^^,^^3^ Intraoperative point-probe Raman spectroscopy systems have also been developed lending rapid tissue characterization and classification to guide surgical procedures based on statistical models produced using machine learning techniques.^4^ For example, instruments have been developed to help reduce instances of positive margins in breast-conserving surgery,^5^ to detect normal brain invaded with cancer cells during glioma surgery,^6^^,^^7^ and to characterize prostate tissue;^8^ they have been integrated in endoscopic procedures to characterize suspicious lesion in the gastrointestinal tract.^9^

The biochemical information conveyed by a Raman measurement consists of a detailed spectral tissue fingerprint conveying information relating to Raman-active vibrational modes (e.g., C─C stretch, ${\mathrm{CH}}_{2}/{\mathrm{CH}}_{3}$ deformations, and vC═C) associated with native biomolecules. The relative contribution of those bonds to a spectrum can be reinterpreted as a relative fraction of lipids (e.g., phospholipids and cholesterol), nucleic acids, proteins (e.g., collagen and porphyrins), and amino acids (e.g., thymine and phenylalanine). Beyond molecular sensitivity, the strengths of Raman spectroscopy include the fact that it does not require the injection of a tracer and that it can be nondestructive and nonionizing, usually interrogating tissue with excitation light in the near-infrared^10^ at low power levels. However, an important limitation of Raman spectroscopy is that the fraction of the signal directly attributable to molecular vibrational information can be several orders of magnitude smaller when compared to background signals (Fig. 1). Background contributions are usually mostly attributable to intrinsic tissue fluorescence although other sources of background associated with instrument response (e.g., fluorescence or Raman signal generated from silica in optical fibers) or bleed-through at laser excitation can negatively impact Raman signal detection.

**Fig. 1:**
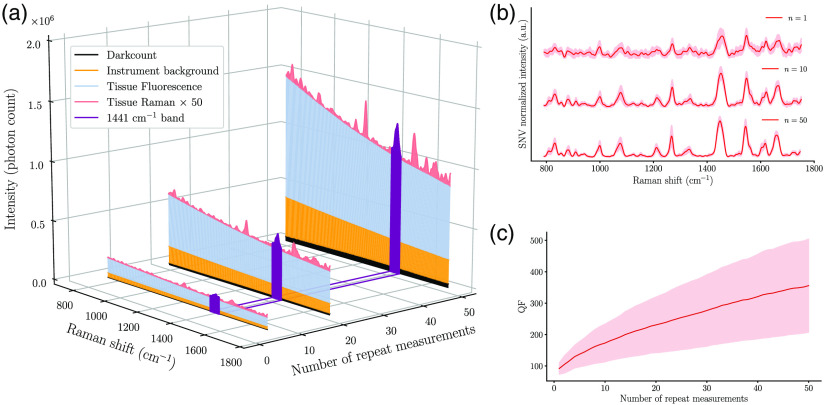
(a) Depiction of the relative proportion of different sources of signal in a human brain measurement made using a Raman spectroscopy system, including dark counts and background (e.g., fluorescence from tissue and optical components). Measurements are shown that were averaged over different number of repeated acquisitions: n=5, 20, and 50. The $1441\;{\mathrm{cm}}^{-1}$ band is highlighted to represent a band typically used to assess spectral quality. For visualization purposes, the Raman signal shown is amplified by a factor of 50. (b) Processed Raman spectra acquired *in vivo* in brain cancer tissue for different numbers of repeat measurements (1, 10, and 50). (c) QF as a function of the number of repeat measurements. In both (b) and (c), the solid line and shaded area represent the average and the standard deviation over 15 spectra acquired at different brain locations, respectively.

An outstanding issue in tissue Raman spectroscopy is that there is no universal approach allowing to quantitatively and automatically establish the spectral quality associated with a Raman measurement. This introduces ambiguities in signal interpretation with no guarantee that the vibrational information is captured at sufficiently high levels of inelastic scattering photon counts for pathology detection. In most experiments reported in the literature, qualitative data quality assessment is made offline based on visual evaluation of the spectra rather than using unbiased quantitative criteria. In fact, it is common to simply assess the presence of relevant tissue Raman bands, including phenylalanine at $1004\;{\mathrm{cm}}^{-1}$, phospholipids at $1087\;{\mathrm{cm}}^{-1}$, tryptophan at $1339\;{\mathrm{cm}}^{-1}$, lipid side chains–amino acids at $1441\;{\mathrm{cm}}^{-1}$, and collagen–nucleic acids at $1659\;{\mathrm{cm}}^{-1}$. However, a quantitative method was developed to filter undesirable skin measurements using principal component analysis to detect and remove outliers in a dataset;^11^ however, this approach is agnostic to the actual biomolecular tissue content.

In biomedical tissue optics applications of Raman spectroscopy reported in the literature, qualitative spectral quality is optimized by tuning the imaging parameters (laser power at the sample, detector exposure time, and number of repeat measurements) all the while ensuring tissue absorption-related heat generation remains sufficiently low not to cause tissue damage. This is especially important for instruments intended to be used in a clinical environment where attaining optimal inelastic scattering photon counts is critical if measurements are to be used to detect subtle changes associated with pathological tissue alterations.

Finding standardized and quantitative manners to assess spectral quality is a complex problem not only because of the observed variability in the relative strength of different Raman bonds across tissue types, but also because biological tissue can be highly heterogeneous in terms of intrinsic fluorescence, absorption (tissue chromophores, e.g., hemoglobin, and pigments, e.g., melanin), and elastic scattering (organelles, cell nuclei, and membranes). As an example, consider measurements made at two different tissue locations having the same concentration of Raman-active biomolecules, but with different levels of background. Then, using identical imaging parameters with the same system at both locations would result in detected Raman photon counts with different stochastic variances from shot noise, potentially resulting in one of the measurements being unable to capture subtle yet important biological Raman peaks (e.g., phenylalanine). These signal quality variations could compromise classification model robustness and performance.

We are presenting the development and in-human validation of a technique that can be used to automatically and unambiguously quantify the shot noise specifically related to the Raman contribution of a spectroscopic measurement, which is the sum of photons from background signals and inelastically scattered Raman photons. The method is applied to *in vivo* human brain data to demonstrate that the resulting Raman signal-to-noise ratio (SNR) can be quantified and used as a surrogate for spectral quality either retrospectively on existing datasets or live during surgical procedures. The new quality metric could be used to establish quantitative thresholds ensuring spectral quality of intraoperative measurements through live automated adjustment of imaging parameters. This could be used to ensure interpatients homogeneity of Raman spectral quality in the scope of clinical studies and trials.

It is well-known that the noise affecting optical measurements made with light sensors [e.g., charged-coupled devices (CCD)] includes at least three important contributions: thermal noise, readout noise, and shot noise (photon noise). For CCD-based detection, thermal noise originates from thermo-generated charges in the depletion region of the chip and readout noise is associated with the measurement of these charges by the readout device. Contrary to those electronic sources of stochastic noise, shot noise is a direct consequence of the particulate nature of light and is common to any photon-detection device. In Raman spectroscopy of biological tissue, thermal and readout noise are usually negligible compared to shot noise because of the large measurement backgrounds.

Shot noise follows a Poisson distribution, but in Raman spectroscopy, it can be approximated as a Gaussian distribution because the overall “fluorescence + Raman” detected light intensity I is always large. This is because significant numbers of Raman photons (relative to the background) can only result if large fluorescence counts are reached. Then, the formula for overall SNR is $I/\sqrt{I}$ (intensity over variance), where I can be modeled as a sum over all detected light contributions, namely background (fluorescence, instrument response, and laser bleed-through), dark counts, and Raman signal. The SNR associated with a measurement can be expressed as the sum of the SNR values associated with each of these individual sources. As a result, the formula quantifying only the Raman SNR—i.e., the shot noise associated only with the Raman component of the signal—within a spectral bin is given by

$$\mathrm{Raman}\;{\mathrm{SNR}}_{j}\approx \sqrt{ntI_{S}}\frac{r_{j}}{\sqrt{r_{j}+a_{j}}},$$ (1)

where j=1 to N is an index labeling the individual spectral bins and N is the number of bins of which a spectrum is composed, usually around 1000 for most spectrometers. Other physical quantities in the formula are the background intensity $a_{i}$, the Raman contribution $r_{i}$, the number of repeat measurements n at each location, the acquisition time t, and the laser power at the sample $I_{S}$.

The hypothesis tested here is that a quality factor (QF) metric can be computed based on the Raman SNR formula and used to quantitatively assess the spectral quality of individual tissue Raman spectra. This will be achieved using an *in vivo* brain dataset acquired using a single-point hand-held Raman spectroscopy probe system published elsewhere.^12^ The dataset consists of 315 *in situ* spectra from 44 brain cancer patients. On average, 7 acquisition points were selected for each patient and n=5 to 10 co-located spectra acquired at each point. The laser power $I_{s}$ (at sample) ranged from 10 to 75 mW and the exposure time was either 50 or 75 ms; both laser power and integration time were recorded to be used in Eq. (2). For each acquisition point, a co-located biopsy sample was analyzed by an expert neuropathologist and assigned one of three labels: normal, cancer, or infiltrated tissue (normal tissue with low density of cancer cells). The ratio of cancer-to-normal samples in the dataset was approximately 1:1.75.

To provide a ground truth in terms of signal quality, all spectra were qualitatively evaluated based on their quality (i.e., presence and relative intensity of standard inelastic scattering peaks expected in tissue) by three independent reviewers (F.D., G.S., and E.L.) using the LabelBox platform (San Francisco, California). Before being presented to each of the reviewers, the spectra were randomly shuffled and their assigned pathology label hidden. Each spectrum was graded on a 1 to 3 scale (higher values corresponding to higher quality) and the sum of all reviewer scores corresponds to the final quality score (qS).^12^ Specific criteria were used such as visual assessment of ubiquitous Raman tissue peaks, including phenylalanine at $1004\;{\mathrm{cm}}^{-1}$, a nucleic acid band at $1082\;{\mathrm{cm}}^{-1}$, the amide III band at $1300\;{\mathrm{cm}}^{-1}$, the ${\mathrm{CH}}_{2}/{\mathrm{CH}}_{3}$ deformation band at $1441\;{\mathrm{cm}}^{-1}$, and the amide I band at $1659\;{\mathrm{cm}}^{-1}$.

In another experiment, 15 *in vivo* brain measurements were made during surgery in one glioblastoma patient to evaluate the impact of n (number or repeat measurements) on the Raman SNR. For this experiment, measurements were made only within an area associated with tumor tissue with a laser power of 30 mW (at sample), an integration time varying between 90 and 600 ms, and n=50. The integration time was automatically determined through an automatic exposure control code automatically adjusting time to ensure at least 50% of the CCD dynamical range was used for each measurement.

The quantitative spectral QF is defined as

$$\mathrm{QF}=\overset{k}{\underset{j=1}{\sum }}\text{Raman }{\mathrm{SNR}}_{j},$$ (2)

where the sum runs over all spectral bins within the Raman bands selected to assess quality (Table 1); for example, see Fig. 1 where the $1441\;{\mathrm{cm}}^{-1}$ band is highlighted. To compute the QF metric, raw spectroscopic data within the *in vivo* brain datasets were preprocessed to separate the Raman contributions $r_{i}$ from the background $a_{i}$ within each spectral bin. Data preprocessing steps detailed elsewhere^12^ included: (1) dark noise subtraction from a measurement with the laser turned off, (2) normalization with the instrument intensity-response correction from a measurement made on a fluorescence standard (SRM 2241, NIST),^13^ (3) background removal using a rolling ball algorithm,^14^ and (4) standard normal variate (SNV) normalization.

**Table 1:** Raman bands considered when computing the QF metric along with associated vibrational modes and families of biomolecules.

Raman band (${\mathrm{cm}}^{-1}$)	Vibrational bonds	Molecular families
1087	C─C stretch	Lipids–DNA
1441	${\mathrm{CH}}_{2}/{\mathrm{CH}}_{3}$ deformation	Lipids–proteins
1553	vC═C–amide II	Proteins
1659	Amide I–vC═C	Lipids–proteins–DNA

The QF metric was then computed for all data points acquired in the scope of the two experiments of the two experiments before SNV normalization. For comparison purposes, the QF was calculated for all four preselected bands in Table 1, but also for the $1441\;{\mathrm{cm}}^{-1}$ band alone, the $1659\;{\mathrm{cm}}^{-1}$ band alone, and the sum of both. Other bands could also be considered, but here we focused on only a few of the most prominent brain tissue Raman bands to demonstrate applicability of the technique.

All spectra were assigned a label associated with the qS metric, either low quality for qS<7 or high quality for qS≥7. Spectra were also each assigned a computed QF value. The QF threshold $\left({\mathrm{QF}}_{\text{thresh}}\right)$ was then varied from ${\mathrm{QF}}_{\text{thresh}}=0$ up to its highest value within the dataset, each time assigning spectra with $\mathrm{QF}>{\mathrm{QF}}_{\text{thresh}}$ as high quality and low quality otherwise. The value ${\mathrm{QF}}_{\text{thresh}}$ was then used as the parameter of a receiver-operator-characteristic (ROC) curve to evaluate the effectiveness of QF to predict spectral quality—i.e., to assess the correspondence between the subset of spectra with $\mathrm{QF}>{\mathrm{QF}}_{\text{thresh}}$ and spectra for which qS≥7. The effect of signal quality on tissue classification performance was assessed using support vector machine (SVM) models with feature selection based on a linear SVM with L1 regularization algorithm.^15^ Model training was done using the Raman spectra associated with normal and cancer tissue based on a fivefold cross-validation with SVM hyperparameters optimized using a grid search. It is standard in the field of machine learning to use a cross-validation procedure because it allows the full dataset to be used during the training phase while ensuring no bias results when selecting the validation set for each fold. An ROC curve analysis was used to compare the performance of two different models trained on spectra with QF>0 and $\mathrm{QF}>{\mathrm{QF}}_{\text{thresh}}$.

Figure 1(b) shows the Raman spectra for the data acquired in one glioblastoma patient with n=50 repeat measurements. Average SNV-normalized spectra and their variance are shown for n=1, 10, and 50, qualitatively highlighting the increase in spectral quality with n. This can be visually assessed based on a decrease of the variance related to shot noise across the spectrum. Figure 1(c) shows the QF metric (for all bands in Table 1 combined) as a function of n, quantitatively demonstrating that the average Raman SNR (over all 15 measurement points) increases like $\sqrt{n}$ as predicted by Eq. (1). The AEC data acquisition algorithm causes the overall photon count within each spectral bin to be approximately constant across measurements. However, the photon count associated with the Raman signal itself varies at different interrogation points, explaining the observed Raman SNR variance in Fig. 1(c).

ROC curves were produced to assess the performance of the QF metric to classify brain Raman spectra as either low (qS<7) or high (qS≥7) qualitative spectral qS. The ROC curves were parameterized by the numerical value of the QF metric and the classification performance were reported in terms of sensitivity (rate of false negatives) and specificity (rate of false positives). Figure 2(a) shows the ROC curves for all considered combinations of Raman bands. The optimal point in the figure (red dot) corresponds to the QF threshold that optimizes both sensitivity and specificity. Using the 1441 and $1659\;{\mathrm{cm}}^{-1}$ bands with QF>145 provided optimal performances with sensitivity and specificity at 89% and 90% respectively. All of the misclassified measurements (approximately 1 out of 10) had a qS score of either 6 for the false positives or 7 for the false negatives—i.e., they were at the margin between high and low quality. Further, visual assessment of all misclassified spectra allowed to determine that they easily could have been classified with a different qS (±1), highlighting a limitation of the qualitative assessment method. Figure 2(b) shows changes in classification performance (cancer vs. normal) when using all normal and cancer spectra (QF>0) compared to using only those with QF>145. The ROC curve parameter value optimizing sensitivity and specificity is shown as a red dot in the figure. Accuracy, sensitivity, and specificity of 72%, 61%, and 75% were obtained for QF>0 while values of 80%, 81%, and 87% were obtained for QF>145, respectively. The cross-validation approach allowed classification model uncertainties to be computed providing information not only on increased model performances for larger QF values but also demonstrated increased model stability. The variability in classification sensitivity and specificity was between 10% and 17% for QF>0 and ranged from 4% to 9% for QF>145.

**Fig. 2:**
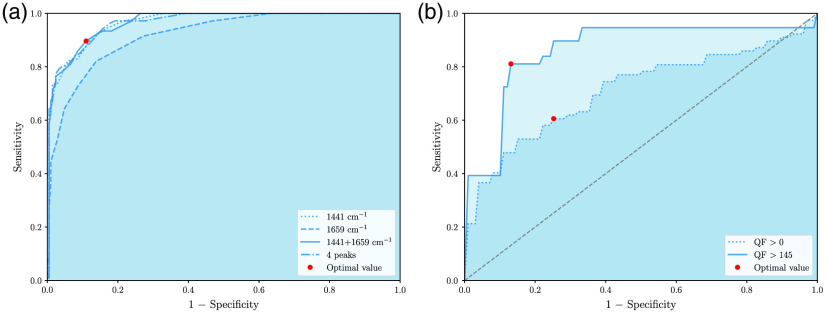
ROC curves showing (a) the correspondence between the qualitative and quantitative spectral quality metrics and (b) the classification performance for different QF thresholds. (a) Each ROC curves were computed for different combinations of Raman bands and are parameterized with the quantitative QF-metric. The qualitative threshold for high spectral quality was qS≥7. The QF value optimizing both sensitivity and specificity is shown as a red dot and corresponds to QF=145. (b) ROC curves for normal versus cancer classification using all spectra from the dataset (QF>0) and only spectra with QF>145. The parameter on the curves optimizing sensitivity and specificity is shown as a red dot.

Figure 3 shows the average spectra (for the 44 patients dataset) for measurements associated with the normal and cancer tissue labels, separated as higher and lower quality using either the qualitative or the quantitative metrics. This shows that the overall Raman signal variance is lowered by selecting spectra with qS≥7 or QF>145. Importantly, the cancer-to-normal samples ratio remained of the same order within the high-quality category as in the full unfiltered dataset, indicating neither of the methods is biased toward a given class when assessing signal quality.

**Fig. 3:**
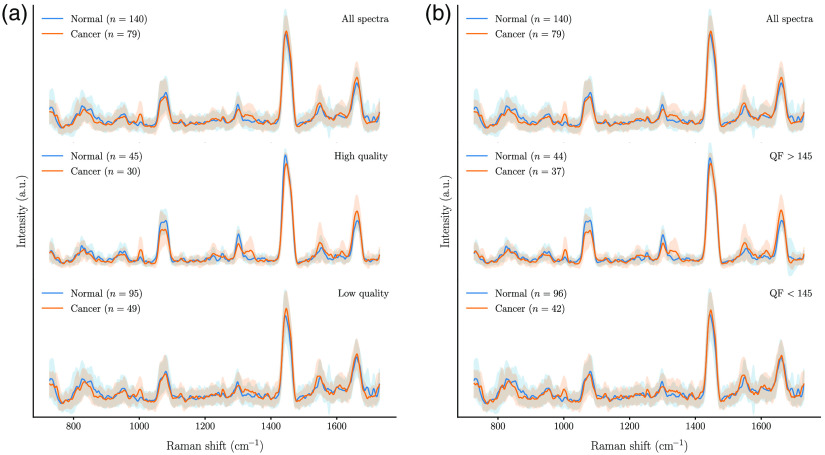
Average spectra for normal and cancer tissue samples classified in terms of spectral quality using: (a) the qualitative qS and (b) the quantitative QF. The top graphs show all spectra independent of spectral quality, the middle graphs are associated with high-quality spectra (qS≥7 or QF≥145) while the graphs at the bottom correspond to low-quality spectra (qS<7 or QF<145).

In most classification approaches based on Raman spectroscopy measurements, interclass differences are small and can be lost in photon noise. Reducing the signal variance due to the shot noise should lead to the development of more robust and generalizable models. This is expected because restraining data to only high QF values would provide the classification algorithms with the opportunity to more efficiently capture biomolecular tissue differences. Of course, classification performances could decrease if the dataset becomes too small because of more stringent imposed spectral quality requirements during the data acquisition process.

The new spectral quality quantification method can be a powerful tool for offline data assessment on existing datasets. However, its main strength resides in its potential use for real-time Raman spectroscopy signal acquisition. For example, an SNR control unit could be added to the acquisition workflow of point-probe systems used for surgical guidance. A particular implementation would require three user-defined input parameters: (1) a predetermined threshold value QF required to capture spectral variations between classes, (2) a fixed laser power value, and (3) the maximum number of spectra $n_{\max}$ incurring no tissue damage. Then, once the integration time is set by the automatic exposure control algorithm, data acquisition would be done n times until either the Raman SNR threshold is reached or until $n=n_{\max}$. This would allow to optimize spectral quality within safety limits imposed by the requirement measurements should have no impact on the tissue beyond transient heating effects.
